# Launching *eLife*, Part 1

**DOI:** 10.7554/eLife.00270

**Published:** 2012-10-15

**Authors:** Randy Schekman, Mark Patterson, Fiona Watt, Detlef Weigel

**Keywords:** publishing, peer review, open access, *eLife*

## Abstract

The new open-access journal *eLife* has launched, making its first content available in PubMed Central. In addition to publishing science of the highest quality, the journal aims to improve both the peer-review process and the presentation of new research results.

The eLife initiative is an unprecedented collaboration between the funders and practitioners of life and biomedical science, aiming to catalyse fundamental change in research communication. Motivated by the tremendous opportunities afforded through digital media, the Howard Hughes Medical Institute, the Max Planck Society and the Wellcome Trust have come together with the research community to create a new and transformative journal, as a first step in the broader eLife project.

The three legs of eLife's initial mission are: to publish outstanding science under an open-access license; to create an unparalleled editorial process that is decisive, fair and efficient; and to fully utilize digital media in the presentation of new research. The guiding principle of the project is to serve the interests of science, scholars and society.

Since the announcement of the project in 2011, we have recruited a world-class community of academic editors to run the journal along with a staff team of experienced professionals. In partnership with a number of different organizations, we have also established much of the essential publishing infrastructure required to meet eLife's objectives.

*eLife* opened for submissions in mid-June, and within two months about 100 submissions had been received, including several exceptional articles that met the standards for scientific excellence required for publication in *eLife*. A key goal for *eLife* is to eliminate unnecessary delays in the publication process, and the fact that the first articles were accepted in August demonstrates that our editorial and peer-review processes are equal to this challenge.

Given that we were able to accept several articles quickly and that the launch of the *eLife* journal website is still several weeks away, we have been considering ways to avoid any unnecessary delays in publishing these articles. To that end, from October 2012, we are listing articles on the eLife website as soon as they are ready to be published and releasing the articles themselves on PubMed Central (PMC), the public archive for life and biomedical science literature at the US National Library of Medicine. The articles are available in full-text HTML and fully formatted PDF versions. They are the final published versions, each one complete with a broadly accessible summary (the *eLife* Digest). Each of the first four articles is also accompanied by an Insight article, which offers the perspective of an expert in the field.

The initial collection of content covers a broad range of topics, reflecting our goal to encompass biomedical and life science from the most basic research through to translational, applied and clinical work. One article identifies a critical signalling molecule involved in the interaction between a primitive eukaryote and a bacterium, which is an important advance in efforts to understand the evolution of multicellularity. This article received substantial publicity after one of the principal authors, Nicole King, presented the work (already accepted for publication in *eLife*) at the Society for Developmental Biology meeting, and then posted the accepted article on her own lab website. The article was downloaded over 1000 times in the first 30 days.

How cells cope with the stress of poorly folded proteins is the theme of a second article, which uncovers how fission yeast deploys the same cellular machinery in an unusual and very different way from other organisms. Another article describes the results of a two-year field trial that demonstrates how a specific group of chemicals released by plants in response to herbivore attack can increase the plants' fitness. And to complete this collection of four articles, a significant advance in aging research is provided by researchers who show that a hormone involved in the response to starvation dramatically increases the lifespan of mice in which it is overexpressed. As well as representing diverse areas of research, importantly, the four articles send a strong signal about the calibre of the science that we seek to publish at *eLife*.

Newly published articles will continue to be added to the list at elifesciences.org over the coming weeks, with links to the content in PMC. But it is at the *eLife* journal website where we will have the most scope to experiment and explore new territory in research communication, beginning with the release of the initial version of the site towards the end the year—part 2 of the *eLife* journal launch.

We hope that these first articles will inspire many more of our colleagues throughout the world to submit their best work to *eLife*, where they will benefit from a constructive and swift publishing experience, a flexible online platform that allows them to tell their research story in full alongside other outstanding work, and the full recognition and attention of peers and interested readers without limit.

We would like to close by thanking our supporters, along with editorial and staff colleagues for their hard work and commitment in getting us this far, and especially the initial authors, who have taken a bold step to get *eLife* off to such a flying start.

